# A Novel Approach to ICD Lead Revision in a Patient with Extensive Vascular Thrombosis

**Published:** 2010-02-01

**Authors:** Sony Jacob, Deepti Bhandare, Anil Mathew, M Salik Jahania

**Affiliations:** 1 Division of Cardiology / Electrophysiology, Department of Internal Medicine, Wayne State University, Detroit, Michigan; 2 Department of Surgery, Division of Cardiothoracic Surgery, Wayne State University, Detroit, Michigan; 3 Department of Internal Medicine, Division of Cardiovascular Diseases, University of Florida, Jacksonville, Florida

**Keywords:** ICD Lead Revision, Extensive Vascular Thrombosis

## Abstract

Chronic extensive thrombosis of the venous system is a commonly encountered problem in end stage renal disease (ESRD) patients undergoing transvenous device implantation, lead extraction or lead revision. We describe a case of an ESRD patient with an implantable cardioverter defibrillator (ICD) that failed to deliver therapy due to lead fracture. Patient needed revision of the ICD lead system, but had extensive axillary-subclavian-superior vena cava occlusion. Patient refused a thoracotomy approach as well as lead extraction as he had a complicated course of lead extraction in the past. We successfully improvised a novel technique to revise the ICD system.

## Introduction

Device implantation related complication rates are higher in ESRD patients than those without renal disease. Obstruction of the upper thoracic venous system is a frequent impediment to device implantation in such patients [1]. Open thoracotomy is an alternative approach but not a viable option in all patients. Femoral approach to lead revision, usually allows the device position in the abdomen or thigh which is known to be uncomfortable [2-4] and may cause high defibrillation threshold (DFT). In our patient, an iliac approach was adopted with emphasis on preserving the preexisting right prepectoral generator position.

## Case Report

A 64 y/o male ESRD patient on hemodialysis, who had a right sided prepectoral ICD placement for secondary prevention of sudden cardiac death, was admitted for syncope. Device interrogation showed episode of polymorphic ventricular tachycardia with the device failed to deliver any therapy. Interrogation also revealed that the superior vena cava (SVC) and right ventricular (RV) coil impedances were > 200 ohms. Chest x-ray showed subclavian crush damage to the RV lead.  Manipulation of the generator header region essentially ruled out the possibility of loose set screw. However, digital pressure on the subclavicular region changed the coil impedances to 98 and 56 ohms for the proximal and distal coils respectively.  However, pacing and sensing thresholds remained unchanged during these maneuvers. An upper thorax venography was performed which revealed occlusion of the SVC, right cephalic, right subclavian and right internal jugular veins with extensive collateralization. The only active arteriovenous dialysis graft was on the left arm and hence any transvenous access from the left axillary subclavian system was not entertained. Moreover, in the past, the patient had a complicated lead extraction and hence he was unwilling to undergo any lead extraction or a revision by open thoracotomy. A novel technique was improvised for the lead revision accommodating the existing right sided ICD system.

## Technique

A right femoral vein access was secured and venography of the right iliac and femoral veins was performed to map the inferior vena cava (IVC) system. Thereafter, a long guide wire (150cm) was passed via the right femoral vein into the iliac vein.  A 25 gauge spinal needle was introduced into the right iliac vein. This was to avoid the possibility of retroperitoneal bleed in case iliac artery gets punctured.  The guide wire and 25 gauge needle were used as fluoroscopic reference to assist the entry of an 18 gauge cook needle into the iliac vein.  A second guide wire was then introduced through the 18 gauge needle into the iliac vein and the IVC using Seldinger technique.  The 18 gauge needle was then removed and 9 French sheath advanced over the guide wire. Both the reference needle and the guide wire were then removed. Once iliac access was established, an extended length active fixation pacing and defibrillation lead (#6947 -100 cm; Medtronic®, Minneapolis, MN, USA) was advanced via the iliac vein into the RV and secured into the mid septum.

**Figure 1:**
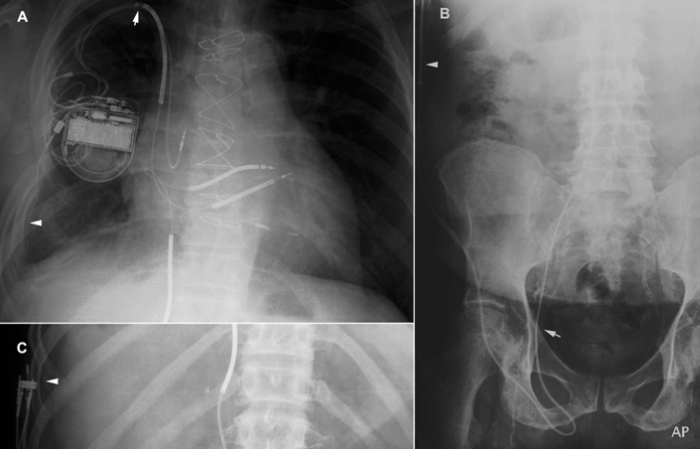
A: A posteroanterior Chest X-ray showing the ICD generator placed in right prepectoral region. Note the new ileofemoral RV defibrillation lead and the preexisting chronic RV defibrillation lead in the RV. Arrow pointing upwards shows the subclavian crush. Arrowhead shows the RV coil with the 25 cm extension in the subcutaneous tunnel. Figure B shows the new RV lead in the right iliac vein, making a U turn in the groin and traversing upward to the chest through the subcutaneous tunnel created. Figure C shows the RV coil in the subcutaneous tunnel and the Y-adaptor extension used to increase the length of the RV lead to reach the device. Note the RV pacing/sensing component and the IVC coil of the lead is capped.

The proximal end of the ICD lead was tunneled subcutaneously in femoral region and looped (u-turn) around (Figure 1B) to provide slack.  Thereafter a subcutaneous tunnel was created from the right groin along the right lateral aspects of the abdominal and chest walls into the right ICD pocket area. A Gore Tunneler with an 8 mm and 10 mm tip was used to create the subcutaneous tunnel and a Penrose drain was used to tunnel the lead. A 25 cm Y-adaptor extension and a 15 cm lead adaptor kit were used to extend the lead to achieve adequate length to reach the device pocket in the right chest. The newly extended lead was then advanced through the tunnel to the ICD pocket, allowing connection with the generator (Figure 1 A-C).

The ventricular sensing and pacing was achieved via the pace/sense lead of the preexisting RV lead. The newly implanted RV coil was used for the defibrillation function. Thus both the lead system was used to reestablish the ICD function. All the nonfunctioning parts of the leads [RV and SVC coils of the chronic lead and the pacing and the IVC (proximal) coil of the new lead] were capped.  Device interrogation with this configuration showed a P wave amplitude of 3 mV, atrial threshold of 1 V at 0.5 milliseconds and impedance of 430 ohms.  R waves were 12 mV; ventricular pacing threshold was 1 V at 0.5 milliseconds with a lead impedance of 720 ohms. Defibrillation threshold (DFT) testing performed per lab protocol demonstrated a DFT of less than 20 Joules (successful defibrillation at 20J x 2 times). Shock impedance of the RV coil was 43 ohms.

The patient had an unremarkable post procedural course and returned to his previous lifestyle. Device interrogation performed 1.5 years later confirmed ICD system integrity to be optimal and unchanged. Moreover, the patient did not experience significant local or systemic complications.

## Discussion

This is the first documentation of ICD lead revision by introduction of the ICD lead from an ileofemoral approach and subcutaneous tunneling from the femoral area to the pectoral region. Previous case reports have described femoral vein access, but the generators were placed in an infradiaphragmatic position [2-4]. Implantation of generator in sites other than pectoral region may encounter high DFTs necessitating implantation of additional subcutaneous coil. By preserving the device's thoracic location, vector related issues and discomfort and movement limitation associated with a femoral or thigh locations were avoided. By creating a loop in the femoral region, enough lead slack was provided. This will give enough room to prevent lead displacement given the range of motion of the hip joint.  Disadvantages of this technique include a long subcutaneous course and use of extensions. Possible complications include retroperitoneal hematoma (from inadvertent iliac artery puncture), peritoneal perforation, lead dislodgement and high DFT values.

Use of multiple extensions of the RV defibrillation lead did not change the impedance. Although, there is a risk of potential damage to the pacing/sensing component of the preexisting RV lead in the future, its use minimized the number of extensions and connectors required. Patient was followed closely and the device was tested monthly for 1.5 years and the pacing sensing lead function was unchanged. However, if it fails in the future, an extension can be made at that time. Additionally the newly introduced SJ4 connector lead (St. Jude Medical, St. Paul, MN) will be a better option in such situations as only one tunneling is needed for the pacing, sensing and shocking leads.

The scarcity of traditional venous access sites and increased risks of open chest surgery in patients with surgically altered cardiac anatomy prompted establishment of the alternate approach described above. The number of devices implanted per year in ESRD patients are increasing and hence the chances of encountering similar situations may be on the rise.
